# Double Cervix with Normal Uterus and Vagina - An Unclassified Müllerian Anomaly

**DOI:** 10.22074/ijfs.2019.5524

**Published:** 2019-01-06

**Authors:** Isabel Lobo Antunes, Cláudia Tomás, Íris Bravo, José Luís Metello, Ana Quintas, Pedro Sá e Melo

**Affiliations:** 1 Gynaecology and Obstetrics Specialist Registrar, Hospital Garcia de Orta, Almada, Portugal; 2 Gynaecology and Obstetrics Consultant, Hospital Garcia de Orta, Almada, Portugal; 3 Gynaecology and Obstetrics Senior Consultant, Hospital Garcia de Orta, Almada, Portugal

**Keywords:** Female Infertility, Female Tract, Müllerian Anomaly, Urogenital Abnormalities

## Abstract

Müllerian anomalies are very common, and a frequent cause of infertility. The most used classification system until now, proposed by the American Society for Reproductive Medicine in 1988, categorizes comprehensively uterine anomalies but fails to classify defects of the cervix or vagina. This is based on a developmental theory that postulates that müllerian duct fusion is unidirectional, beginning caudally and extending cranially, which does not account for isolated cervical or vaginal defects. More recently, the European Society of Human Reproduction and Embryology has developed a consensus, which allows for independent cervical anomalies. We present a case of a 39-year-old woman with secondary infertility, found to have a cervical duplication in an anteroposterior disposition, which puts into question the principles of embryology formerly known, but supports the theory that development happens in a segmentary fashion.

## Introduction

 Female genitourinary tract malformations are extremely common, being found in around 5.5% of the general population and 8% of infertile women, specifically affecting as many as 25% of women with infertility due to miscarriage (1). The real prevalence may be even higher, considering most of them will go undiagnosed either for being asymptomatic or because of having no access to methods for accurate diagnosis. The spectrum of these malformations is enormous, and although there have been several attempts to catalogue them-the most utilized until recently being the classification by the American Society for Reproductive Medicine (ASRM) from 1988 (2), which included mostly uterine anomalies-it is still necessary to extend the list for a more complete record. Over the years reports of types of malformations have arose, not included in this classification system, predominantly of associated or isolated cervical and vaginal anomalies. We present a case of a cervical duplication with a normal uterus and normal vagina, but with an anteroposterior disposition of the cervix, which supports the theory that isolated segment defects may occur. This case questions the embryology theory that has supported the ASRM classification for decades.

## Case Report

**Fig.1:**
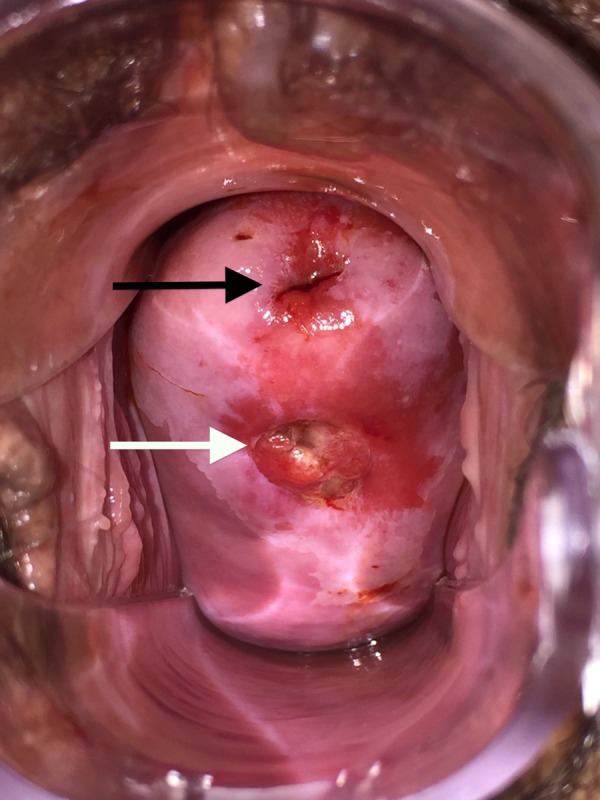
Speculum examination showing anterior (black arrow) and posterior (white arrow) cervical OS.

**Fig.2:**
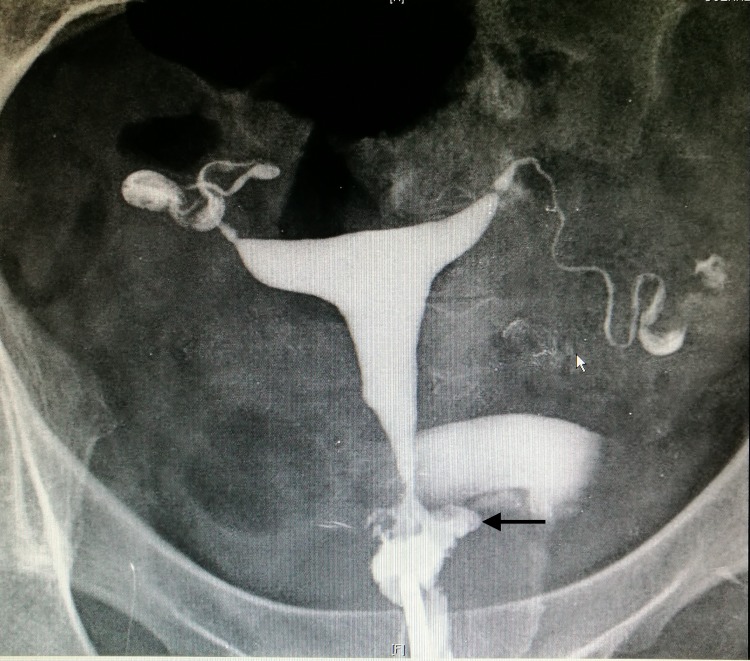
Hysterosalpingogram showing a normal uterine cavity with contrast extravasation through a posterior cervical canal (black arrow).

**Fig.3:**
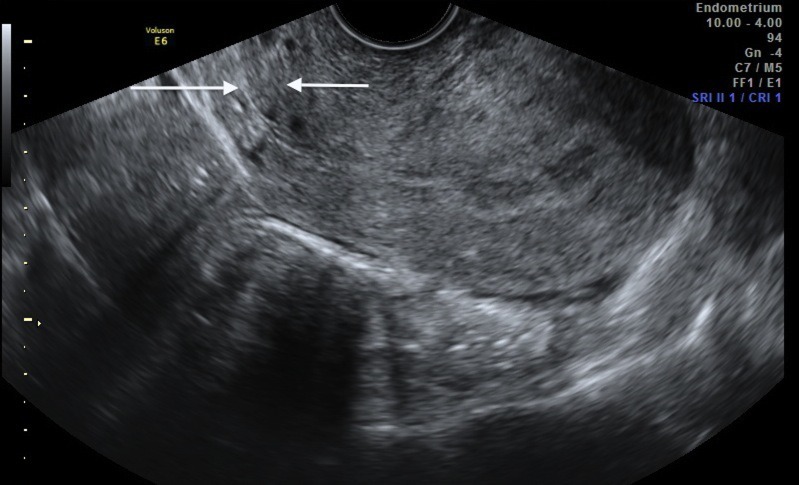
Transvaginal ultrasound displaying one internal cervical os (black arrow) and two cervical canals (white arrows).

 A 39 year-old woman was referred to our institution due to secondary infertility. Menarche was at 14 years of age, with regular cycles and slight dysmenorrhoea. She had experienced a term caesarean section 8 years prior due to failure to progress, and had been trying to get pregnant for 3 years. Her past medical history was unremarkable. On gynaecological examination external genitalia and vagina were normal; two cervical orifices in an anteroposterior disposition were clearly visualized (Fig .1)-this was confirmed with curetting of the posterior canal, which revealed “normal endocervical mucosa”, excluding other pathologies such as uterovaginal/cervicovaginal fistulae. Menstrual blood was observed exiting both cervical orifices. Hysterosalpingography (HSG) revealed a normal uterine cavity and tubes, although contrast was visualized exiting the posterior endocervical canal (Fig .2). Transvaginal ultrasound revealed a normal retroverted uterus, with one internal cervical OS and two endocervical canals diverging from it in an anteroposterior arrangement (Fig .3). Because both these exams did not suggest a uterine cavity defect, we chose not to pursue with further tests such as magnetic resonance imaging (MRI) or hysteroscopy, having to subject the patient to bothersome and invasive testing that would not alter clinical conduct. Consent form was obtained and completed by participant.

## Discussion

An extremely rare müllerian malformation is described, which questions classical knowledge of developmental embryology. An extensive literature search was conducted revealing only a few similar cases (3, 4), one in a 4-month infant with other multiple malformations, and another with a side-by-side disposition of the cervix. Only one other case anatomically similar to ours is depicted in the literature (5).

The aetiology for most of the congenital anomalies of the female genital tract is unknown. The importance of normal embryological development lays in its reproductive consequences, and also in concomitant urological abnormalities (which are more common with more proximal defects) and in quality of life (for possible dysmenorrhoea or dyspareunia in obstructive defects). In 1988 the ASRM attempted to classify these anomalies (2), however it documents only the more common uterine anomalies, not including rarely occurring cervical or vaginal defects (6). Nevertheless, until recently it has been the most commonly used classification for congenital anomalies.

Two main theories, both described in 1960s, are the foundations for the classification system of the ASRM. The first theory, described by Crosby and Hill (7), suggests that uterine development is a result of müllerian duct fusion between the 11th and the 13th weeks of embryonic life, beginning caudally and progressing cranially; this process is then followed by septal reabsorption, which begins at any point of fusion and moves in either or both directions. The downside of this unidirectional theory is that it does not account for lower segment defects with normal upper segments, as is the case of vaginal/cervical duplications with normal uteri. The second theory, argues that müllerian duct fusion is initiated in the middle portion, at the uterine isthmus, and proceeds simultaneously in a cranial and caudal direction, and that the septal reabsorption follows a similar bidirectional pattern, with complete uterus formation independently from the formation of cervix and vagina (8). This theory, which seems to encompass defects not explained by the first, still does not justify the existence of a middle segment isolated defect, as in our case.

Acién et al. (8) advocated that in fact the müllerian ducts do not contribute to the formation of the vagina, instead the vaginal walls are formed by cells from the wolffian ducts and are then covered by cells of the müllerian tubercle. Therefore the processes of fusion and reabsorption of the müllerian ducts may affect: i. Both converging and diverging portions (superior and inferior uterine segments), ii. Just one of them, or iii. Even just a small specific area, giving rise to segmentary defects.

More recently, the European Society of Human Reproduction and Embryology (ESHRE) developed an anatomy-based consensus on congenital anomalies of the female genital tract and its related clinical significance, in a comprehensive and accessible system. Here, cervical and vaginal anomalies are categorized into independent sub-classes (9), and a normal uterus can be associated with either an abnormal cervix or an abnormal vagina, or both. Therefore, this system encompasses “segmentary defects”, and supports Acién’s theory. Yet another classification system, this time by El Saman et al. (10), attempts to classify müllerian duct anomalies based on both embryological development and a “treatment-based” categorization, also including segmentary defects, allowing for a more comprehensive approach to congenital anomalies.

## Conclusion

Embryology of the female genital tract is not completely understood, as the mechanism of müllerian development is more complex than previously described. Acién’s segmentary theory is currently the one that best explains segment malformations as the one presented here. This theory puts into question our decade-long understanding of embryological development of the female reproductive system, and supports the classification system of müllerian anomalies of ESHRE. The fact that in our case the two cervices were displayed in an anteroposterior fashion also calls into question the fact that development may not always occur in a side-by-side manner.
